# Pharmacokinetics and Excretion Study of *Lycium* *barbarum* Polysaccharides in Rats by FITC-Fluorescence Labeling

**DOI:** 10.3390/foods10112851

**Published:** 2021-11-18

**Authors:** Hui Xia, Chao Yang, Beijia Zhou, Huali Tang, Ligang Yang, Wang Liao, Guiju Sun

**Affiliations:** Key Laboratory of Environmental Medicine and Engineering of Ministry of Education, Department of Nutrition and Food Hygiene, School of Public Health, Southeast University, Nanjing 210009, China; huixia@seu.edu.cn (H.X.); wenzhengwuguan@yeah.net (C.Y.); 220193581@seu.edu.cn (B.Z.); tanghl220@yahoo.com (H.T.); yangligang2012@163.com (L.Y.); wangliao@seu.edu.cn (W.L.)

**Keywords:** *L. barbarum* polysaccharides, fluorescein isothiocyanate, pharmacokinetic, excretion

## Abstract

A high-performance gel permeation chromatography fluorescence detection (HPGPC-FD) method combined with fluorescein isothiocyanate (FITC) labeling was established for the microanalysis of *L. barbarum* polysaccharides (LBP). The calibration curves linear over the range of 0.2–20 µg/mL in rat plasma, and 0.25–500 μg/mL in urine and feces samples with correlation coefficients greater than 0.99. The inter-day and intra-day precisions (RSD, %) of the method were under 15% with the relative recovery ranging from 84.6% to 104.0% and the RSD ranging from 0.47% to 7.28%. The concentration–time curve of LBP-FITC in plasma following intragastric administration at 100, 50 and 25 mg/kg well fitted to a nonlinear model. LBP-FITC slowly eliminated from plasma according to the long half-lives (t_1/2_ = 31.39, 38.09, and 45.76 h, respectively) and mean retention times (MRT_0–t_ = 18.38, 19.15 and 20.07 h, respectively; AUC_0–∞_ = 230.49, 236.18 and 242.57 h, respectively) after administration of LBP-FITC at doses of 100, 50, and 25 mg/kg, respectively. After intragastric administration at 50 mg/kg for 72 h, the concentration of LBP-FITC in urine and feces was 0.09 ± 0.04% and 92.18 ± 3.61% respectively; the excretion rate of urine was the highest in 0–4 h period and decreased continuously in 4–24 h period. The excretion rate of feces was the highest in 4–10 h, 48.28 ± 9.349% in feces within 4–10 h, and decreased rapidly in 10–24 h. The present study showed that LBP was absorbed as its prototype and most proportion of LBP was excreted from feces, indicating a long time remaining in intestine.

## 1. Introduction

Pharmacokinetics is to study the internal process of drugs, that is, the quantitative study of the absorption, distribution, metabolism, and excretion of drugs in organisms [1]. Establishing a reliable and reproducible quantitative analysis method is one of the keys to study pharmacokinetics. At present, the commonly used pharmacokinetic analysis methods include chromatography [2,3,4,5,6], spectroscopy [7,8,9], immunology, and microbiology [10,11,12], etc. The specific analysis method should be based on the chemical structure, physical and chemical properties, instrument conditions, and reference literature of the drug. So far, pharmacokinetics has been gradually and wildly used in the study of absorption, metabolism, and distribution of nutrients in vivo studies [13,14].

Lycium barbarum (*L. barbarum*), which is used in traditional medicine to maintain health as a nutraceutical, is popular in China and other Asian countries. Among *L. barbarum* extracts, *L. barbarum* polysaccharides (LBP) isolated from *L. barbarum* fruit have been responsible for the biological activities of *L. barbarum*. As a naturally occurring chemical, LBP is the water-soluble glycoconjugates with a molecular weight from 10–2300 kDa [15]. Due to their various pharmacological effects, including anti-oxidation, anti-aging, immune regulation, improvement of gut microbiota and reproductive protection [16,17,18,19,20,21], LBP has been widely exploited in healthy foods and medicine in China. Although the comprehensive pharmacological studies of LBP have been performed, there is still only limited information on the pharmacokinetics and excretion study of this compound in vivo given their high molecular size. Therefore, a detailed knowledge of its mechanism is important for its biological activities.

Due to the lack of micro quantitative detection technology, the pharmacokinetic study based on LBP is seriously restricted. Recently, techniques based on the detection of fluorescence have been employed in drug microanalysis due to their specificity, sensitivity, and low detection threshold [22]. Fluorescein isothiocyanate (FITC) is a common fluorescent probe in fluorescence immunoassay [23], which is commonly used for fluorescent labeling of proteins. FITC is the most commonly used tracer which can conjugate in other types of biomaterials or biomolecules for imaging [24,25,26,27]. FITC is widely used for protein labeling due to the high reactivity of isothiocyanate groups (N=C=S) with amino groups [28]. LBP is polymerized by mannose, rhamnose, glucose, galactose, xylose, and polypeptide or protein (containing -NH2 or -NH-group) [29]. Modification of the free amino groups with FITC yielded soluble copolymers having covalently bound fluorescein and glycosyl groups [30]. We have successfully synthesized a FITC-labeled LBP according to the method of high-performance gel permeation chromatography fluorescence detection (HPGPC-FD) method [8], and the molecular weight of a fluorescent labeling product of polysaccharide (LBP-FITC) did not change much compared with LBP [31,32].

In this study, we designed and synthesized a fluorescent labeling product of polysaccharide using FITC as fluorescent probe. The absorption and elimination characteristics of LBP in animals were clarified by studying the pharmacokinetics and concentration of LBP in blood and excreta. It is of great significance to accurately grasp the pharmacokinetic information of LBP in vivo and the potential factors, which is conducive to the further exploration for their pharmacological activities.

## 2. Materials and Methods

### 2.1. Chemicals and Reagents

LBP was purified by *Sevage* method [32], and the preparation method of LBP has been published [29,32]. The content of polysaccharide and protein in LBP was 94.9% and 2.8% respectively.

Fluorescein isothiocyanate (FITC) (purity > 99.0%) was purchased from the American Sigma Corporation. Sodium bicarbonate, sodium dihydrogen phosphate, disodium hydrogen phosphate, and sodium chloride were obtained from Sinopharm Chemical Reagent Co., Ltd. (Shanghai, China). Anhydrous ethanol was procured by Wuxi Yasheng Chemical Co., Ltd. (Wuxi, Jiangsu, China). Ethylenediamine was obtained from Jinan Reagent Factory (Jinan, China). A 0.45-μm microporous membrane was procured by Hangzhou Fuqiang Chemical Instrument Co., Ltd. (Hangzhou, China). Ultrapure water was obtained from a Milli-Q water purification system (Millipore, Billerica, MA, USA). All other chemicals were of analytical grade.

### 2.2. Animals

Male Sprague-Dawley (SD) rats with 180 ± 20 g of body weight were obtained from Shanghai Jiesijie Experimental Animal Co., Ltd. (Animal License No.: SCXK (Shanghai)-2013-0006). The rats were fed in the animal laboratory of School of Public Health, Southeast University (Room temperature 21 ± 1 °C with relative humidity 60–100% and 12-h dark-light cycle). Enough tap water and normal chow were provided ad libitum. All rats were acclimated in the laboratory for 1 week prior to the experiment.

All animal experiments were conducted in accordance with the “Principles of laboratory animal care” of NIH and the protocol for animal study of Animal Management Committee and Animal Ethical Committee of Jiangsu Province, and approved by Animal Experimental Ethical Committee of Southeast University (No. 2015-1025-009).

### 2.3. Instrumentation

Fluorescence spectrophotometer (Cary eclipse) was purchased from Varian China Co., Ltd. (Palo Alto, CA, USA). HPLC (Agilent 1260), fluorescence detector (Agilent 1260), gel chromatography column (SB-804 HQ) were obtained from Agilent Technologies Co., Ltd. (Santa Clara, CA, USA). Gel chromatography column (SB-804 HQ) was purchased from Agilent Technology Co., Ltd. (Santa Clara, CA, USA). Centrifuge (L-550) was purchased from Hunan centrifuge instrument Co., Ltd. Micro vortex mixer (WH-3) was obtained from Shanghai Huxi Analytical Instrument Factory Co., Ltd. (Shanghai, China). Portable high-speed homogenizer (S10) was obtained from Ningbo Xinzhi Biotechnology Co., Ltd. (Ningbo, China). Magnetic stirrer (85-1) was procured from Jintan medical instrument factory. Vacuum freeze dryer (FD-1) was procured from Beijing Boyaikang Experimental Instrument Co., Ltd. (Beijing, China). PH meter (PHS-25) was purchased from Shanghai Instrument & Electronics Co., Ltd. (Shanghai, China). Electronic analytical balance (BSA124S) was obtained from Mettler Toledo, Switzerland (Zurich, Switzerland). Ultrasonic cleaner (TM-010) was obtained from Kunshan Ultrasonic Instrument Co., Ltd. (Kunshan, China). 

### 2.4. Establishment of Quantitative Analysis Method for LBP-FITC

#### 2.4.1. Fabrication of LBP-FITC

LBP (500 mg) was dissolved in 25 mL pure water, and pH was adjusted to 8.0 with 0.5 mol/mL NaHCO_3_, then 25 mg FITC was added. After stirring for 24 h under room temperature and being kept in dark environment, the reaction solution was filtered and then anhydrous ethanol was added to the filtrate until the final concentration of ethanol was 80% (*v*/*v*). There was precipitation, and the supernatant was discarded after centrifugation. The precipitate was re-dissolved by adding water, and then precipitated again by absolute ethanol repeating for three times. Then the precipitate was washed repeatedly with anhydrous ethanol until no fluorescence absorption was found in the supernatant. After freeze-dried precipitation, LBP-FITC was obtained [31]. The excitation wavelength Ex and emission wavelength Em of LBP-FITC were 495 nm and 518 nm with fluorescence spectrum. The average molecular weight of LBP-FITC was 4920 Da detected with HPGPC.

#### 2.4.2. Preparation of LBP-FITC Standard Solution

Standard stock solution preparation: 100 mg of LBP-FITC sample was accurately put into a 100 mL volumetric flask, and then PBS solution was added to dilute to the scale. Total of 1 mg/mL of LBP-FITC standard stock solution was prepared and stored at 4 °C as standby.

LBP-FITC series concentration standard solution preparation: we accurately measured the appropriate amount of LBP-FITC standard stock solution, and then prepared 0.2, 1, 2, 4, 10, 20, 50, 100, 500, 1000 μg/mL LBP-FITC standard solution with PBS solution.

### 2.5. Sample Collection

#### 2.5.1. Urine and Fecal Samples

The rats ate and drank freely before the experiment, and the experiment was carried out three days after adaptation. The rats were placed in a metabolic cage and fed in a single cage one day before execution, collecting urine and feces meanwhile. Urine samples were centrifuged at 4000 r/min for 10 min, and the supernatant was filtered by 0.45 μm filter membrane and transferred to EP tube for test or stored in −70 °C refrigerator for testing. Fecal samples were collected with self-sealed bags, weighed and ground after natural air drying, and then put into EP tube for the later usage or stored in −70 °C refrigerator for standby.

#### 2.5.2. Plasma Sample

After anesthetizing with ether, the blood samples were put into the anticoagulant tube treated with EDTA as anticoagulant, centrifuged at 4 °C and 3500 r/min for 10 min. The upper plasma was put into the EP tube, which could be used immediately or stored in −70 °C refrigerator for standby.

### 2.6. Sample Determination

#### 2.6.1. Determination Conditions

According to our previous study [31], the excitation wavelength Ex and emission wavelength Em of LBP-FITC are 495 nm and 518 nm with fluorescence spectrum analysis, respectively.

#### 2.6.2. Plasma Samples Determination

Each plasma sample (100 μL) was spiked with 0.01 mol/L PBS to 1.4 mL, vortexed for 1 min, and then let to stand for 10 min. The suspension was centrifuged at 12,000 r/min for 10 min, and we determined the fluorescence intensity of the supernatant.

#### 2.6.3. Urine Samples Determination

Treated urine sample (20 μL) was mixed with 0.01 mol/L PBS to quantify to 1.8 mL, and vortexed for 1 min, and then allowed to stand for 10 min. The suspension was centrifuged at 12,000 r/min for 10 min, and then the fluorescence intensity of the supernatant was determined.

#### 2.6.4. Fecal Samples Determination

Treated fecal samples (0.05 g) were put into a 2 mL EP tube, and 0.01 mol/L PBS was added to quantify to 1.5 mL. The mixture was vortexed for 5 min and centrifuged at 12,000 r/min for 10 min. Then 500 μL supernatant was transferred to the EP tube and 0.01 mol/L PBS was added to quantify to 1.8 mL. The suspension was centrifuged at 12,000 r/min for 10 min, and the fluorescence intensity of the supernatant was determined.

### 2.7. Fabrication of Standard Curve

#### 2.7.1. Plasma Samples

Blank plasma (100 μL) and 25 μL LBP-FITC series standard solution were accurately measured. Seven plasma samples with LBP-FITC concentration (0.2, 0.4, 0.8, 2, 4, 10, and 20 μg/mL) were prepared and analyzed.

#### 2.7.2. Urine Samples

The calibration curve standard samples were prepared by spiking 20 μL LBP-FITC series standard solution into 20 μL of rat blank plasma at final concentrations of 0.25, 1.25, 6.25, 62.5, 125, 250, and 500 μg/mL.

#### 2.7.3. Fecal Samples

Total of 500 μL supernatant of each blank fecal extract and 750 μL of LBP-FITC series standard solution were accurately measured to prepare fecal samples with concentration of 5, 25, 37.5, 75, 150, and 300 μg/mL respectively.

Blank biological samples with the same volume of PBS instead of LBP-FITC standard solution were prepared. The serial concentrations of LBP-FITC in plasma, urine, and feces were used as the abscissa (X), and the difference between the fluorescence intensity measured and that of blank plasma, urine, and feces was taken as the ordinate (Y). A standard curve (linear regression equation) was obtained using the weighted least squares method (W = 1/x^2^). The preparation process was carried out in a dark environment.

### 2.8. Method Validation

Total of 100 μL, 20 μL, and 500 μL of rat blank plasma, urine, and feces were respectively used, and LBP-FITC series standard solution was accurately added.

#### 2.8.1. Precision

In order to evaluate the precision of the method, five samples of quality control (QC) with three concentration levels of low, medium, and high were prepared. According to the corresponding standard curve of the day, the determination concentration of QC sample was calculated, and the precision of the method is obtained by comparing with the prepared concentration. Five replicate analyses were performed on each sample for five consecutive days. The intra-day and inter-day relative standard deviations (RSD) were calculated.

#### 2.8.2. Stability

The stability index evaluates the stability of the test object at each step of the method, which generally includes short-term, long-term, freeze-thaw, and autosampler stabilities. The short-term, autosampler, and long-term stabilities were evaluated by measuring QC samples at room temperature for 24 h and at −20 °C for 15 days, respectively. The freeze-thaw stability was measured by three freeze-thaw cycles on consecutive days.

#### 2.8.3. Recovery Rate and Matrix Effect

In order to evaluate the recovery rate, five samples of each QC samples (low, medium, and high concentrations) were analyzed. The relative recovery rate was calculated as the percentage of drug concentration after regression to the actual drug concentration. The matrix effect was measured by comparing the peak areas of the unextracted standard biological samples with those of neat samples at an equivalent concentration. The assessment of a relative matrix effect was performed based on direct comparison of the responses (peak areas) of the analyte spiked into extracts originating from six different lots (sources) of biofluids. The variability in these responses, determined as RSD (%), was considered as the measure of relative matrix effect for a given analyte [33,34,35].

### 2.9. Plasma Pharmacokinetics

#### 2.9.1. Administration Method

LBP-FITC samples were prepared into 100, 50, and 25 mg/mL solutions with normal saline.

Eighteen male Sprague Dawley rats were randomly divided into three dose groups (n = 6 in each group). The rats were administrated through intragastric administration with three dosages (100 mg/kg, 50 mg/kg, and 25 mg/kg) after fasting and freely accessing to water for 12 h.

#### 2.9.2. Sampling Method

About 0.5, 1, 2, 3, 4, 6, 8, 10, 12, 24, 36, and 48 h after intragastric administration, blood samples (0.3 mL) were collected from tail vein into K2-EDTA anticoagulant tubes before intragastric administration. The blood samples were centrifuged at 4 °C, 3500 R/min for 10 min within 1 h. The plasma was collected by micropipette into the EP tube for experiment or stored in the refrigerator at −70 °C.

### 2.10. Excretion Study

#### 2.10.1. Administration Method

LBP-FITC was accurately weighed and prepared into 50 mg/mL solution with normal saline.

Six male Sprague Dawley rats were used for the experiment after three days of adaptation. LBP-FITC was given orally by gavage with a dose of 50 mg/kg.

#### 2.10.2. Sample Collection and Processing

Before administration, six rats were placed in a metabolic cage with fasting and freely accessing to water for 12 h, the blank urine and feces were collected at the same time. After single intragastric administration, urine and feces were collected at 0–4 h, 4–10 h, 10–24 h, 24–48 h, 48–72 h respectively. Urine and feces were collected with a centrifuge tube wrapped with tin foil, and the volume of urine was recorded. The urine was stored in a refrigerator at −70 °C after being filtered by a 0.45-μm filter membrane. The feces were dried naturally and the weight was recorded.

### 2.11. Sample Determination

According to our previous study [31], the excitation wavelength Ex and emission wavelength Em of LBP-FITC were 495 nm and 518 nm, respectively. Detailed methods on determination of plasma, urine, and fecal were described as Section 2.6.

### 2.12. Data Processing

#### 2.12.1. Pharmacokinetic Data Processing

The pharmacokinetic parameters, including maximal plasma concentration (C_max_), the time to peak plasma concentration (T_max_), the area under the plasma concentration-time curve (AUC), the elimination half-life (t_1/2_), and the mean residence time (MRT), were analyzed using DAS (Drug and Statistics) software (Version 2.0).

The C-T data of drug time curve were calculated automatically by DAS 2.0 (Drug and Statistics) statistical software. The C-T data of 6 rats at each time point were processed in batches, and the statistical moment analysis of non-compartment model was performed to obtain the pharmacokinetic parameters. The experimental results were expressed as mean ± SD.

#### 2.12.2. Data Processing of Excretion Experiment

According to the standard curve of the day, the concentrations of LBP-FITC in urine and feces of rats were calculated respectively. The concentrations of LBP-FITC in urine and feces of each rat after administration were listed respectively, and the cumulative excretion amount, excretion percentage, and excretion rate were calculated respectively. The cumulative excretion curve of LBP-FITC in urine and feces was drawn according to the mean value and standard deviation. Origin software was used to process the data.

## 3. Results and Discussion

### 3.1. Establishment and Validation of Quantitative Analysis Method

#### 3.1.1. Standard Curve Establishment

The samples obtained by adding LBP-FITC standard solution and PBS with the same volume were determined, and the fluorescence intensity was recorded. The measured fluorescence intensity minus the fluorescence intensity of blank biological sample was taken as the ordinate (Y) and the FBP-FITC concentration (μg/mL) as the abscissa (X).

Blank plasma, urine and feces samples of rats were used to establish the LBP-FITC standard curve, and all correlation coefficients of the linear regression equation of each biological sample were greater than 0.99, which indicated that the linear equation could meet the requirements of pharmacokinetic study.

#### 3.1.2. Precision

According to the QC sample, the precision of the experiment was calculated. The experimental results of the QC sample of LPB-FITC were in line with the relevant requirements for biological sample determination. The specific data are shown in Table 1. The intra-day relative standard deviation (RSD) of the concentration of 0.2 μg/mL in rat plasma was 7.28%, 6.90%, 5.37%, 4.88%, and 4.92%, and the inter-day RSD was 7.15%; the intra-day RSD of 2 μg/mL concentration was 3.45%, 4.01%, 4.92%, 2.67%, and 5.39%, and the inter-day RSD was 4.83%; the intra-day RSD of 20 μg/mL was 1.86%, 2.24%, 3.28%, 2.48%, and 1.97%, and the inter-day RSD was 2.62%. As mentioned above, the intra- and inter-day precision values (RSD) of QC samples with all different concentration levels were less than 15%. The results show that the method used in the present study is with good precision.

The intra-day RSD of 0.25 μg/mL concentration in rat urine was 4.26%, 5.90%, 5.37%, 4.88%, and 4.92%, and the inter-day RSD was 7.16%; the intra-day RSD of 50 μg/mL concentration was 1.45%, 2.01%, 1.92%, 2.67%, and 1.39%, respectively, and the inter-day RSD was 1.83%; the intra-day RSD of 500 μg/mL concentration was 0.86%, 1.04%, 0.58%, 0.68%, and 0.47%, respectively, and the inter-day RSD was 0.78%. As shown, the intra- and inter-day RSDs of low, medium, and high levels are relatively small (less than 15%) and the precision of the method was proved as good.

The intra-day RSD of 5.0 μg/mL concentration in rat feces was 3.68%, 3.92%, 6.21%, 4.86%, 3.90%, and the inter-day RSD was 6.12%; the intra-day RSD of 50.0 μg/mL concentration was 1.66%, 1.91%, 2.04%, 1.69% and 1.12%, and the inter-day RSD was 2.86%. The inter-day RSD of 300.0 μg/mL concentration was 0.89%, 1.53%, 1.24%, 1.08%, and 1.37%, respectively, and the inter-day RSD was 1.74%. The intra- and inter-day RSDs of low, medium, and high levels were relatively small (less than 15%). The results showed that the precision of this method was good.

#### 3.1.3. Stability

The fluorescence intensity was measured after 24 h storage at room temperature, three times of freeze-thaw, and cryopreservation at −20 °C for 15 d. The stability of LBP-FITC was evaluated by analyzing the above three conditions, as shown in Table 2. For LBP-FITC, the intra- and inter-day precision values (RSD, %) were both lower than 5%. These results, which were within the acceptable criteria for precision, proved that this method was reliable for the quantitative analysis of LBP-FITC in rat biological samples.

#### 3.1.4. Recovery and Matrix Effect

By spiking standard solutions into the processed blank plasma, urine, and feces sample, three kinds of solutions with low, medium, and high concentrations were prepared. The results of matrix effect and biological sample recovery are shown in Table 3. It could be seen from the table that the inter-group recoveries of plasma samples were between 84.6% and 104.0%, with RSD ranging from 1.97% to 7.28%; the intra-group recoveries of low, medium, and high concentration plasma samples were between 91.6% and 100.2%, with RSD of 4.58%, 3.82%, and 2.04%, respectively.

The inter-group recovery rate of urine samples was between 95.5% and 100.8%, with RSD range from 0.47% to 5.90%; and intra-group recovery of low, medium, and high concentration urine samples was between 97.6% and 99.3%, with RSD of 1.42%, 1.64%, and 0.38%, respectively.

As shown in Table 3, the inter-group recovery rate of low, medium, and high concentration fecal samples was between 94.6% and 100.6%, with RSD ranging from 0.89% to 6.21%; the intra-group recovery of fecal samples was between 98.2% and 99.4%, with RSD of 2.78%, 0.52%, and 1.00%, respectively. Although several studies [8,36,37] reported biological samples to be deproteinized would affect the recovery rate of polysaccharides, our results indicated that extraction recovery of this method was consistent, reproducible, and acceptable.

### 3.2. Pharmacokinetics Study

#### 3.2.1. Plasma Concentration-Time Data

LBP-FITC was given to rats by gavage at the dose of 100, 50, and 25 mg/kg. After administration, the blood concentration of LBP-FITC was measured at 0, 0.5, 1, 2, 3, 4, 6, 8, 10, 12, 24, 36, and 48 h. The data were shown in Table 4.

#### 3.2.2. Mean Plasma Concentration–Time Curve

**Figure 1:**
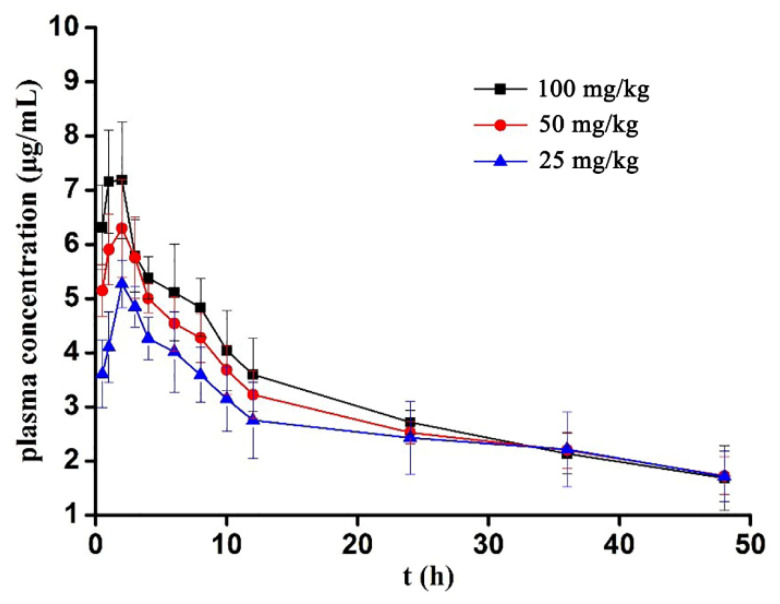
Mean-plasma concentration–time profile of LBP-FITC after intragastric administration of LBP-FITC to rats.

The mean plasma concentration–time curves of LBP-FITC in the experimental rats are shown in Figure 1. After single intragastric administration of LBP-FITC of different doses to rats, the average drug time curve was made by plotting the blood drug concentration and corresponding detection time points. The results showed that the plasma concentration–time curves were all nonlinear with intragastric administration of different doses. The concentration–time curves of bioactive polysaccharides in animal plasma were mostly confirmed to be two-compartment model, but their pharmacokinetics parameters showed significant differences due to different physicochemical characteristics, administration methods, and dosages [38]. Consistent with our results, mussel polysaccharide MA showed a nonlinear characteristic with the dose range of 25–100 mg/kg in rats [38]. 

#### 3.2.3. Pharmacokinetic Parameters

The pharmacokinetic data of LBP-FITC were analyzed by DAS 2.0 software. The non-compartment model based on statistical moment theory was used to fit the blood concentration time data of LBP-FITC in rats. The main pharmacokinetic parameters were calculated as shown in Table 5. After a single oral administration of LBP-FITC at doses of 100, 50, and 25 mg/kg, the t_1/2_ was 31.39 ± 14.64, 38.09 ± 9.43, 45.76 ± 4.57 h, respectively; the AUC_(0–t)_ was 151.09 ± 15.10, 141.25 ± 12.02, 128.21 ± 27.64 mg/L.h, respectively; the AUC_(0–∞)_ was 230.49 ± 73.26, 236.18 ± 35.08, 242.57 ± 64.09, respectively; the MRT_(0–t)_ of 0–t was 18.38 ± 1.01, 19.15 ± 0.84, 20.07 ± 1.49 h, respectively; the maximum C_max_ was 7.44 ± 0.72, 6.56 ± 0.51, 5.27 ± 0.44 mg/L; the T_max_ was 2.00 ± 0.63, 2.33 ± 0.52, 2 ± 0.00 h, respectively. In this study, T_max_ of the high, medium, and low-dose groups were all small, while T_1/2_ value was higher, which indicated that LBP-FITC absorbed rapidly in rats, but the elimination is slow, which may be related to the properties of polymer. LBP is polymerized by glucose, mannose, and galacturonic acid, and the molecular weight of LBP is about 5 kDa [29]. Kaneo et al. [39] revealed that fluorescein-labeled polysaccharide (dextrans) with a molecular weight (Mw) lower than 20 kDa showed rapid elimination from the blood after intravenous injection. But this correlation was not exited in the present study. The slow elimination from the blood could be explained directly by the mode of intragastric administration. Consistent with our results, Lin et al. [4] revealed the elimination half-life of Ophiopogon japonicus polysaccharide FOJ-5 with relative molecular weight of 5 kDa in rat plasma was longer after intragastric administration than that of intravenous injection.

**Figure 2:**
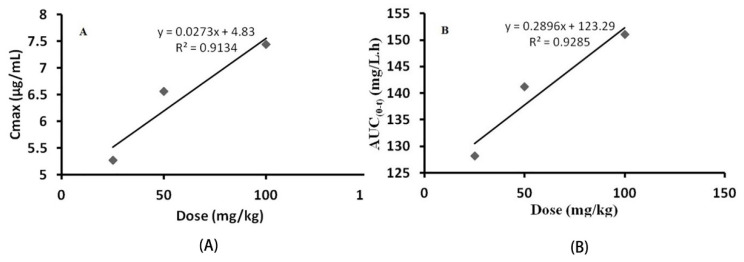
Relationship between C_max_ (**A**), AUC_(0–t)_ (**B**) and different dose after oral administration of LBP-FITC to SD rats.

The pharmacokinetic parameters of C_max_ and AUC_(0–t)_ were used to plot three dose levels of LBP-FITC. As shown in Figure 2, the C_max_ and AUC_(0–t)_ of each dose group were positively correlated with the dosage, indicating that LBP-FITC presented linear pharmacokinetic characteristics in experimental rats. Similar results that pharmacokinetics exhibited an appreciable dose dependency have been observed in other studies on the pharmacokinetics of polysaccharide [39,40,41].

### 3.3. Excretion Study

**Figure 3:**
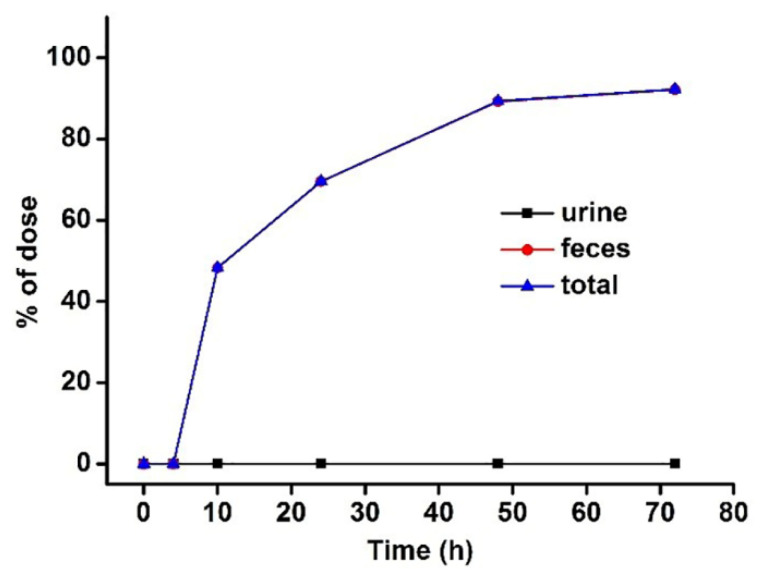
The percentage of cumulative amount of LBP-FITC excreted in rat urine and feces in dose after a single i.g. dose of 50 mg/kg LBP-FITC.

**Figure 4:**
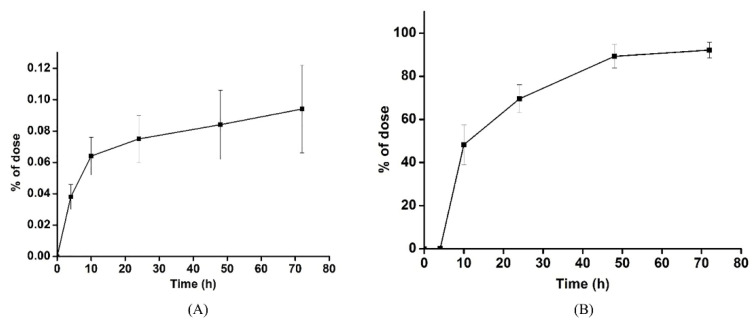
Cumulative amounts of LBP-FITC excreted in urine (**A**) and feces (**B**) after an oral dose of 50 mg/kg to rats.

**Figure 5:**
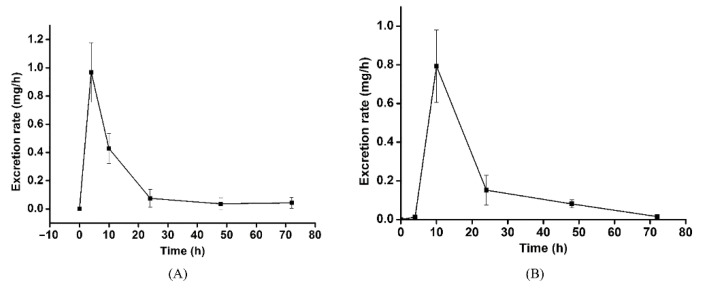
Excretion rate of LBP-FITC excreted in urine (**A**) and feces (**B**) after an oral dose of 50 mg/kg to rats.

After a single dose of 50 mg/kg LBP-FITC, the average cumulative excretion amount and percentage of LBP-FITC in urine and feces at different time periods were shown in Table 6. As shown, the cumulative excretion rate of LBP-FITC in urine and feces was 0.09 ± 0.04% and 92.18 ± 3.61% respectively after a single oral administration of 50 mg/kg LBP-FITC for 72 h. As shown in Figure 3 that the cumulative excretion rate of urine and feces reached 92.27% within 72 h after a single oral administration of 50 mg/kg LBP-FITC, with fecal excretion being the main one. The results showed that the absorption rate of LBP was not high in vivo. The average curves of cumulative excretion of urine and feces in the percentage of administration dose are shown in Figure 4A,B. The cumulative excretion rate of LBP-FITC in urine and feces at different time periods is shown in Table 7. After intragastric administration of LBP-FITC, the excretion rate of urine was the highest in 0–4 h period and decreased continuously in 4–24 h period. However, the cumulative excretion rate in urine accounted for only 0.094 ± 0.036% in 0–72 h. The excretion rate of feces was the highest in 4–10 h, 48.28 ± 9.349% in feces within 4–10 h, and decreased rapidly in 10–24 h. Excretion rate of LBP-FITC excreted in urine and feces after an oral dose of 50 mg/kg to rats is showed in Figure 5A,B. Retention of a large amount of LBP in intestine suggested it may play potential effects of LBP on intestinal which were proved in some studies reported [42,43].

## 4. Conclusions

LBP-FITC has the property of fluorescence generation, and the quantitative analysis method of LBP-FITC in vivo has been successfully established, and the method was applied to study the pharmacokinetics of LBP-FITC in vivo. The pharmacokinetic parameters of LBP-FITC after single oral administration of LBP-FITC in SD rats were obtained. The half-life of LBP-FITC in rats was long with slow elimination. The excretion study showed that most of LBP-FITC was excreted from urine and feces with the proportion of 92.274% of LBP-FITC after 72 h of administration, in which 92.18% of LBP-FITC was excreted from feces. LBP, as a kind of polysaccharides, may mainly act on the intestinal tract due to its difficulty to be absorbed.

## Figures and Tables

**Table 1:** Inter-day and intra-day precision of LBP-FITC in blank rat plasma, urine, and feces (*n* = 5).

Biological Sample	Theoretical Concentration (μg/mL)	Inter-Day, RSD (%)					Intra-Day, RSD (%)
		1	2	3	4	5	
Plasma	0.2	7.28	6.9	5.37	4.88	4.92	7.75
	2.0	3.45	4.01	4.92	2.67	5.39	4.83
	20.0	1.86	2.24	3.28	2.48	1.97	2.62
Urine	0.25	4.26	5.9	5.37	4.88	4.92	7.16
	50.0	1.45	2.01	1.92	2.67	1.39	1.83
	500.0	0.86	1.04	0.58	0.68	0.47	0.78
Feces	5.0	3.68	3.92	6.21	4.86	3.9	6.12
	50.0	1.66	1.91	2.04	1.69	1.12	2.86
	300.0	0.89	1.53	1.24	1.08	1.37	1.74

**Table 2:** Stability in rat plasma, urine, and feces (*n* = 5).

Biological Sample	Theoretical Concentration (μg/mL)	24 h at Room Temperature		Repeated Freeze-Thaw Test for 3 Times		Frozen at −20 °C for 15 Days	
		Measured Concentration (μg/mL)	RSD (%)	Measured Concentration (μg/mL)	RSD (%)	Measured Concentration (μg/mL)	RSD (%)
Plasma	0.2	0.196 ± 0.004	4.90	0.194 ± 0.005	4.98	0.191 ± 0.003	3.80
	2.0	1.89 ± 0.02	2.07	1.93 ± 0.04	4.03	1.96 ± 0.03	3.24
	20.0	19.17 ± 0.34	3.74	19.03 ± 0.24	2.72	19.49 ± 0.17	1.87
Urine	0.25	0.246 ± 0.004	4.61	0.244 ± 0.005	4.98	0.247 ± 0.003	4.78
	50	48.89 ± 0.82	1.07	49.93 ± 0.74	1.83	49.96 ± 0.83	1.34
	500	498.17 ± 3.34	0.74	496.03 ± 2.24	0.72	498.49 ± 3.17	0.89
Feces	5.0	4.92 ± 0.14	3.64	4.86 ± 0.12	4.02	4.96 ± 0.13	3.82
	50.0	48.89 ± 0.22	2.11	46.93 ± 0.34	5.36	48.96 ± 0.23	4.64
	300.0	287.63 ± 0.34	3.28	279.08 ± 0.24	2.72	292.44 ± 0.31	2.81

**Table 3:** Inter- and intra-group matrix effect and extraction recovery for LBP-FITC in rat plasma, urine, and feces (*n* = 5).

Biological Sample	Theoretical Concentration (μg/mL)	Inter-Group										Intra-Group	
		1		2		3		4		5			
		Rate (%) (M ± SD)	RSD (%)	Rate (%) (M ± SD)	RSD (%)	Rate (%) (M ± SD)	RSD (%)	Rate (%) (M ± SD)	RSD (%)	Rate (%) (M ± SD)	RSD (%)	Rate (%) (M ± SD)	RSD (%)
Plasma	0.2	99.0 ± 7.9	7.28	96.0 ± 6.1	6.90	99.5 ± 7.8	5.37	104.0 ± 5.6	4.88	97.5 ± 7.2	4.92	100.2 ± 4.6	4.58
	2.0	93.2 ± 3.8	3.45	87.1 ± 3.5	4.01	84.6 ± 2.9	4.92	95.2 ± 3.4	2.67	94.3 ± 5.2	5.39	91.6 ± 4.4	3.82
	20.0	98.4 ± 1.2	1.86	96.9 ± 1.8	2.24	98.9 ± 1.6	3.28	94.6 ± 0.9	2.48	97.2 ± 1.9	1.97	98.3 ± 2.8	2.04
Urine	0.25	99.2 ± 4.8	4.28	100.0 ± 5.1	5.90	98.4 ± 5.6	5.37	100.8 ± 4.8	4.88	98.0 ± 6.4	4.92	98.4 ± 1.4	1.42
	50	97.7 ± 1.7	1.45	95.5 ± 1.6	2.01	97.4 ± 1.9	1.92	99.8 ± 1.7	2.67	95.8 ± 1.6	1.39	97.6 ± 1.5	1.64
	500	99.5 ± 0.5	0.86	99.1 ± 0.7	1.04	99.4 ± 0.4	0.58	98.8 ± 0.6	0.68	99.7 ± 0.7	0.47	99.3 ± 0.4	0.38
Feces	5.0	99.6 ± 4.78	3.68	97.2 ± 4.20	3.92	94.6 ± 2.72	6.21	99.2 ± 6.40	4.86	100.6 ± 3.18	3.90	98.2 ± 2.72	2.78
	50.0	96.5 ± 1.64	1.66	96.3 ± 1.56	1.91	96.2 ± 1.86	2.04	95.6 ± 1.34	1.69	95.4 ± 1.06	1.12	98.3 ± 0.50	0.52
	300.0	99.6 ± 1.05	0.89	99.3 ± 1.21	1.53	99.4 ± 1.50	1.24	98.9 ± 1.02	1.08	99.8 ± 1.24	1.37	99.4 ± 0.98	1.00

**Table 4:** Plasma concentration (μg/mL) of LBP-FITC after intragastric administration of LBP-FITC to rat at dosage of 100 mg/kg, 50 mg/kg, and 25 mg/kg (*n* = 6).

Concentration	100 mg/kg						50 mg/kg						25 mg/kg					
Time(h)	1	2	3	4	5	6	1	2	3	4	5	6	1	2	3	4	5	6
0	0	0	0	0	0	0	0	0	0	0	0	0	0	0	0	0	0	0
0.5	5.176	5.722	6.454	6.425	6.69	7.426	4.933	5.082	5.358	5.527	4.333	5.632	3.514	4.050	4.180	4.132	3.121	2.645
1	5.305	7.451	7.084	7.679	7.463	7.975	4.724	6.331	6.174	6.564	5.666	5.963	4.391	4.641	4.578	4.427	3.519	3.054
2	5.160	7.543	7.624	7.706	6.878	8.228	4.623	6.494	6.634	7.214	6.078	6.747	4.741	5.976	5.403	5.194	5.404	4.898
3	6.085	6.343	5.407	5.023	5.219	6.677	5.902	6.056	5.401	4.98	5.146	7.021	4.496	5.184	5.315	4.413	5.038	4.635
4	5.677	5.158	5.321	5.186	4.93	6.001	4.897	5.235	5.111	4.666	4.789	5.336	4.193	4.237	4.872	3.938	4.524	3.816
6	5.555	4.09	5.231	4.844	4.394	6.578	4.206	4.004	4.859	4.758	4.143	5.282	3.988	3.320	4.855	4.672	4.273	2.974
8	4.045	4.713	4.88	4.784	4.875	5.714	3.981	3.651	4.546	4.256	4.254	4.967	3.728	3.019	4.026	3.947	3.949	2.874
10	3.582	3.11	3.726	4.987	4.027	4.832	3.515	2.897	3.669	4.034	3.812	4.185	3.413	2.478	3.640	3.773	3.213	2.376
12	2.903	3.923	2.76	4.557	3.777	3.658	2.808	2.682	3.528	3.879	3.221	3.241	3.051	2.566	3.449	3.296	2.642	1.511
24	2.592	2.631	2.756	2.612	2.551	3.146	2.349	2.476	2.743	2.581	2.756	2.248	3.026	2.126	2.996	2.551	2.638	1.239
36	1.884	1.858	2.507	1.748	2.161	2.668	1.964	1.869	2.721	2.511	2.108	2.046	3.018	1.992	2.671	2.498	2.088	1.034
48	1.592	1.161	2.065	1.499	1.118	2.689	1.362	1.558	2.014	2.123	1.964	1.337	2.094	1.423	1.941	2.039	1.901	0.892

**Table 5:** Pharmacokinetic parameters of LBP-FITC after intragastric administration of LBP-FITC to rats at dosage of 100 mg/kg, 50 mg/kg, and 25 mg/kg.

Parameter	Unit	100 mg/kg		50 mg/kg		25 mg/kg	
		Mean	SD	Mean	SD	Mean	SD
C_max_^a^	mg/L	7.44	0.72	6.56	0.51	5.27	0.44
T_max_^b^	h	2	0.63	2.33	0.52	2	0
AUC_(0–t)_^c^	mg/L.h	151.09	15.10	141.25	12.02	128.21	27.64
AUC_(0–∞)_^d^	mg/L.h	230.49	73.26	236.18	35.08	242.57	64.09
MRT_(0–t)_^e^	h	18.38	1.01	19.15	0.84	20.07	1.49
t_1/2_^f^	h	31.39	14.64	38.09	9.43	45.76	4.57
CLz/F ^g^	L/h/kg	0.47	0.12	0.22	0.03	0.11	0.04
Vz/F ^h^	L/kg	18.96	3.66	11.57	2.16	7.29	2.38

^a^ Cmax: maximum plasma concentration; ^b^ Tmax: maximum plasma time; ^c/d^ AUC: area under the plasma concentration–time curve, which is the parameter reflecting the total amount of drug entering the blood circulation; ^e^ MRT: mean residence time, which refers to the average time of drug molecules remaining in the body; ^f^ t_1/2_: half-life, represents the time required to eliminate half of the drug concentration in plasma after the drug distribution is balanced in vivo, which is an important parameter to express the speed of drug elimination in vivo; ^g^ CL: plasma clearance, which is the parameter for estimating the drug elimination rate from the body; ^h^ V_z_/F: apparent volume of distribution.

**Table 6:** Mean cumulative amounts of LBP-FITC excreted in urine and feces after an oral dose of 50 mg/kg to rats (Mean ± SD, *n* = 6).

Time	Urine		Feces	
	Amount (μg)	% of Dose	Amount (μg)	% of Dose
0–4	3.87 ± 0.04	0.038 ± 0.008	0.05 ± 0.02	0.052 ± 0.018
0–10	6.44 ± 0.06	0.064 ± 0.012	4.81 ± 1.21	48.28 ± 9.349
0–24	7.49 ± 0.09	0.075 ± 0.015	6.94 ± 1.18	69.53 ± 6.483
0–48	8.36 ± 0.13	0.084 ± 0.022	8.88 ± 1.12	89.24 ± 5.487
0–72	9.38 ± 0.23	0.094 ± 0.036	9.23 ± 0.50	92.18 ± 3.609

**Table 7:** Urinary and feces excretion rate of LBP-FITC after an oral dose of 50 mg/kg to rats.

Time (h)	Δt (h)	Urinary		Feces	
		Excretion Amount (mg)	Excretion Rate (mg/h)	Excretion Amount (mg)	Excretion Rate (mg/h)
0	0				
4	4	3.87 ± 0.06	0.968 ± 0.211	0.05 ± 0.02	0.013 ± 0.005
10	6	2.57 ± 1.24	0.428 ± 0.107	4.76 ± 1.12	0.793 ± 0.187
24	14	1.05 ± 0.89	0.075 ± 0.064	2.13 ± 1.08	0.152 ± 0.077
48	24	0.87 ± 1.03	0.036 ± 0.043	1.94 ± 0.51	0.081 ± 0.021
72	24	1.02 ± 0.92	0.043 ± 0.038	0.35 ± 0.13	0.015 ± 0.005

## Data Availability

Not applicable.
